# RNA-Seq Profiling in Chicken Spleen and Thymus Infected with Newcastle Disease Virus of Varying Virulence

**DOI:** 10.3390/vetsci11110569

**Published:** 2024-11-15

**Authors:** Xiaoquan Wang, Xiaolong Lu, Mingzhu Wang, Qiwen Zhou, Xiyue Wang, Wenhao Yang, Kaituo Liu, Ruyi Gao, Tianxing Liao, Yu Chen, Jiao Hu, Min Gu, Shunlin Hu, Xiufan Liu, Xiaowen Liu

**Affiliations:** 1 Key Laboratory of Avian Bioproducts Development, Ministry of Agriculture and Rural Affairs, Yangzhou University, Yangzhou 225000, China; wxq@yzu.edu.cn (X.W.); 008328@yzu.edu.cn (X.L.); 19352670779@163.com (M.W.); 13862344808@163.com (Q.Z.); bioyusy@outlook.com (X.W.); 008465@yzu.edu.cn (W.Y.); 007622@yzu.edu.cn (R.G.); 2693040819ltx@gmail.com (T.L.); biochenyu@hotmail.com (Y.C.); hijiao@yzu.edu.cn (J.H.); gumin@yzu.edu.cn (M.G.); slhu@yzu.edu.cn (S.H.); 2 Jiangsu Co-Innovation Center for Prevention and Control of Important Animal Infectious Diseases and Zoonosis, Yangzhou University, Yangzhou 225000, China; liukaituo@yzu.edu.cn; 3 Jiangsu Key Laboratory of Zoonosis, Yangzhou University, Yangzhou 225000, China; 4 Joint International Research Laboratory of Agriculture and Agri-Product Safety of Ministry of Education of China, Yangzhou University, Yangzhou 225000, China

**Keywords:** Newcastle disease virus, vaccine strain, RNA-seq, chicken, immune organ

## Abstract

Newcastle disease virus (NDV), known as avian paramyxovirus-1, poses a significant threat to poultry production worldwide. Vaccination currently stands as the most effective strategy for Newcastle disease control. However, the mesogenic vaccine strain Mukteswar has been observed to evolve into a velogenic variant JS/7/05/Ch during poultry immunization. Here, we aimed to explore the mechanisms underlying virulence enhancement of the two viruses. Pathogenically, JS/7/05/Ch mediated stronger virulence and pathogenicity in vivo compared to Mukteswar. Comparative transcriptome analysis revealed 834 differentially expressed genes (DEGs), comprising 339 up-regulated and 495 down-regulated genes, in the spleen, and 716 DEGs, with 313 up-regulated and 403 down-regulated genes, in the thymus. Gene Ontology (GO) analysis indicated that these candidate targets primarily participated in cell and biological development, extracellular part and membrane composition, as well as receptor and binding activity. Furthermore, Kyoto Encyclopedia of Genes and Genomes (KEGG) analysis unveiled a substantial portion of candidate genes predominantly involved in cellular processes, environmental information processing, metabolism, and organismal systems. Additionally, five DEGs (TRAT1, JUP, LPAR4, CYB561A3, and CXCR5) were randomly identified through RNA-seq analysis and subsequently confirmed via quantitative real-time polymerase chain reaction (qRT-PCR). The findings revealed a marked up-regulation in the expression levels of these DEGs induced by JS/7/05/Ch compared to Mukteswar, with CYB561A3 and CXCR5 exhibiting significant increases. The findings corroborated the sequencing accuracy, offering promising research directions. Taken together, we comprehensively evaluated transcriptomic alterations in chicken immune organs infected by NDV strains of diverse virulence. This study establishes a basis and direction for NDV virulence research.

## Simple Summary

This study conducted RNA-seq to analyze virulence enhancement of a velogenic Newcastle disease virus (NDV) variant, JS/7/05/Ch, compared to the mesogenic vaccine strain Mukteswar. Transcriptome analysis in infected chicken immune organs revealed significant differences in gene expression. These genes were involved in various cellular and biological processes. Five candidate differentially expressed genes were further validated by quantitative real-time PCR, confirming the accuracy of RNA-seq results. The findings provide insights into NDV virulence research and may guide future studies on virus pathogenicity.

## 1. Introduction

Newcastle disease (ND) is caused by virulent forms of avian paramyxovirus of serotype 1 (APMV-1) and holds significant global economic importance [1,2]. The causative agent, Newcastle disease virus (NDV), can be classified into four main pathogenicity groups: avirulent, low pathogenic (lentogenic), intermediate pathogenic (mesogenic), and highly pathogenic (velogenic) strains. Among these, virulent NDV strains typically induce a highly contagious and economically devastating viral disease [3,4]. NDV possesses a negative-sense, single-stranded RNA genome with two membrane glycoproteins: hemagglutinin-neuraminidase (HN) and the fusion (F) protein [5]. These membrane proteins are essential for viral infection and pathogenicity [2,4]. The HN protein, which serves multiple functions, plays a crucial role during various stages of infection. It is responsible for neuraminidase and receptor-binding activities, as well as facilitating the fusion activity of the F protein, which is responsible for virus–cell and cell–cell fusion [6,7,8,9,10]. Importantly, the HN protein has been extensively demonstrated to contribute to NDV virulence and pathogenicity. The complete substitution or single mutations in the HN protein can potentially alter the virulence of NDV [11,12]. Structural domains of the HN protein crucial for replication and species-specific phenotypes include the cytoplasmic tail and stalk domain [13]. Additionally, the 5′ UTR of HN mRNA plays a vital role in viral replication and pathogenicity in vivo [14].

Vaccination stands as the primary method for controlling ND at present [15]. Mukteswar, a mesogenic vaccine strain, has traditionally been administered in poultry during emergencies due to its robust immunogenicity [16]. However, the immune pressure resulting from frequent vaccination has led to the emergence of viral variants [17]. One such virulent genotype III NDV, JS/7/05/Ch, was isolated, sharing over 99% genomic identity with Mukteswar. Further assays revealed that JS/7/05/Ch had evolved from Mukteswar and exhibited heightened virulence and pathogenicity, attributed to mutations in the HN protein [16,18]. The mutant HN protein of JS/7/05/Ch significantly influences the biological activities of NDV in vitro and in vivo [18]; meanwhile, the mutant HN protein facilitates cytopathic damage and programmed cell death by attenuating the activation of NF-κB in NDV-infected cells [19]. NDV HN is implicated in viral infection, yet its interactions with host proteins are unexplored. To identify host proteins differentially interacting with NDV HN, we employed mass spectrometry analysis to identify a host protein vimentin that exhibited diverse interaction with HN proteins from Mukteswar and JS/7/05/Ch [20]. Despite extensive research on NDV pathogenicity and mechanisms, the host immune response post-infection remains elusive.

Next-generation sequencing-based Digital Gene Expression (DGE) tag profiling has been employed to investigate changes in gene expression, a technique widely utilized in basic scientific research, elucidating disease mechanisms, identifying biomarkers, and facilitating drug discovery [21]. This study aims to delineate the differential host immune responses to NDV infection in chickens via RNA-seq. By processing and comparing sequencing data, quantifying gene expression levels, analyzing differentially expressed genes between samples, conducting enrichment analysis on these genes, and selecting representative genes for validation, we aim to establish a molecular basis for further understanding the increased virulence of NDV.

## 2. Materials and Methods

### 2.1. Animals, Cells, and Viruses

Specific pathogen-free (SPF) chicken embryos and chickens were purchased from Beijing Merial Company (Beijing, China) and Jinan Speifului Poultry Technology Co., Ltd. (Jinan, China), respectively. Chicken embryo fibroblast cells (CEF) from 10-day-old SPF chicken embryos were grown in Dulbecco’s Modified Eagle Medium (DMEM) with 10% fetal bovine serum (FBS). The mesogenic vaccine strain Mukteswar (GenBank ID: JF950509.1) and its variant NDV strain JS/7/05/Ch (GenBank ID: FJ430159.1) were provided by our laboratory. 

### 2.2. Virulence Determination 

The virulence indexes of NDV were assessed using various methods. The mean death time (MDT) and 50% egg infectious dose (EID_50_) assay were performed in 9–10-day-old SPF chicken embryos. The 50% tissue culture infective dose (TCID_50_) was determined in CEF cells. Additionally, the intracerebral pathogenicity index (ICPI) and intravenous pathogenicity index (IVPI) assays were conducted in SPF chickens. All these assays were carried out in accordance with the standard procedures for ND [22].

### 2.3. Animal Experiments

Mukteswar and JS/7/05/Ch were chosen for challenge experiments involving SPF chickens. In our preliminary studies, we examined different infection doses to assess the pathogenicity of Mukteswar and JS/7/05/Ch in chickens through intravenous injection. We found significant differences in how these viruses affected the chickens at doses of 10^4^, 10^5^, and 10^6^ EID_50_/0.1 mL. JS/7/05/Ch caused rapid illness by the 3rd day and mortality by the 5th day, with symptoms worsening as the dose increased. In contrast, Mukteswar only led to mild respiratory symptoms in the later stages of infection (10th day) at these doses. At 10^7^ EID_50_/0.1 mL, the pathogenicity and mortality rates of JS/7/05/Ch were comparable to the 10^6^ EID_50_/0.1 mL dose and may reach the infection threshold, while Mukteswar induced more severe symptoms by the 8th day, indicating that the higher dose lessened the difference in their pathogenicity. The most distinct difference in pathogenicity was seen at 10^6^ EID_50_/0.1 mL, which we chose as the standard infection dose for this study.

**Figure 1:**
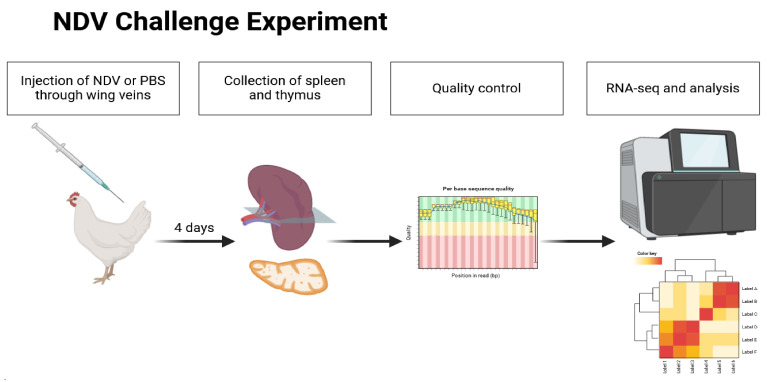
Experimental design strategy in vivo. SPF chickens were injected with NDV and PBS through wing veins. On the 4th day post-infection, the two infected groups were dissected, and the spleen and thymus were collected for subsequent sample quality control, RNA-Seq, and analysis. The schematic representation is created with Biorender.com.

Nine 30-day-old SPF chickens were randomly divided into three groups and housed in separate rooms. Subsequently, each group of three chickens was inoculated with NDVs at 10^6^ EID_50_/0.1 mL through intravenous injection. Concurrently, a control group was established, and an equivalent volume of PBS was injected. On the 4th day post-infection, the two infected groups were dissected, and the spleen and thymus were collected on an ultra-clean platform. The organs from each group, weighing 0.2–0.3 g, were combined in a 2 mL cryotube, immediately subjected to 10 min of liquid nitrogen treatment, and subsequently frozen at −70 °C. The repetitive organs were mixed and then sent to Ruibo Biotechnology Co., Ltd. (Guangzhou, China) for further sample processing, library construction, and sequencing (Figure 1). The rest of the organs were used for subsequent viral titer assessments. Additionally, the remaining spleen and thymus samples were processed through standard histology, encompassing fixation in neutral formalin, dehydration, paraffin embedding, and sectioning followed by baking. The slides were deparaffinized, stained with hematoxylin and eosin, and scrutinized under a light microscope for histopathological changes, with observations documented through photography.

### 2.4. RNA Detection, Library Construction, and Sequencing 

Total RNA was isolated from thymus and spleen tissues using the TransZol Up Plus RNA Kit (TransGen, Beijing, China) according to the instruction, and the extracted total RNA underwent detection using the Agilent 2200 nucleic acid analyzer (Santa Clara, CA, USA). Subsequently, the extracted total RNA underwent Oligo (dt) enrichment to generate polyA RNA, followed by RNA fragmentation treatment. The next steps involved reverse transcription synthesis of the cDNA library, ligation of the DNA sequencing linker, and library amplification through PCR. The amplified library then underwent deep sequencing, and the final step involved image recognition to acquire RNA sequence information. The protocols for library construction and sequencing were facilitated by Ruibo Biotechnology Co., Ltd. (Guangzhou, China).

After acquiring raw data (Raw Data), a series of processing steps were undertaken, including filtering, de-joining, and handling low-quality reads. Subsequently, the sequencing quality was assessed to derive high-quality data (Clean Data), which was then compared to the reference genome. The Tophat2 software (Baltimore, MD, USA) was employed for the comparative analysis of the sequencing data against the reference genome. A comprehensive evaluation encompassed factors such as sequencing data coverage area, coverage depth, read distribution results, transcript coverage, homogeneity, and saturation. Default parameters included -read-mismatches = 2 (allowing two mismatches) and -read-gap-length = 2 (permitting two gaps).

### 2.5. Screening of Differentially Expressed Genes Between Samples

For the identified genes of small sample size, the EdgeR method was employed to calculate gene expression levels and analyze the differentially expressed genes among the samples. Sample selection was based on difference multiples (|log2FoldChange| > 1) and significance levels (q-value < 0.001). Gene expression levels were computed using Reads Per Kilobase per Million (RPKM) values, determined by the formula:

$$\mathrm{RPKM}=\frac{total\;exon\;reads}{mapped\;reads(millions)\times exon\;length(KB)}$$

### 2.6. Inter-Sample Correlation Analysis

The Pearson correlation coefficient (r) is utilized to evaluate the linear correlation between two variables. The closer the absolute value of r is to 1, the more pronounced the similarity in expression patterns between samples. A positive r value indicates a positive correlation in expression patterns, while a negative value signifies a negative correlation. Correlation analysis can, to a certain extent, assess the reliability of sequencing results and offer a comprehensive measure of the differences between samples.

### 2.7. Functional Enrichment Analysis of Biological Pathways for Differentially Expressed Genes

The biological pathway analysis relies on the Kyoto Encyclopedia of Genes and Genomes (KEGG) biological pathway database “http://www.genome.jp/” (accessed on 12 January 2024), which conducts analyses of gene collections within the context of complex regulatory networks. Enrichment analysis serves as a crucial method for studying biological functions. The functional enrichment analysis of genes in the KEGG pathway involves two main steps. Firstly, KEGG pathway annotation provides information on signal transduction and disease pathways for differentially expressed genes, offering background details for gene pathway and functional research. Subsequently, calculations are performed with *p* < 0.05 serving as the significance threshold to identify statistically significant signal transduction and disease pathways relative to the background. This approach helps obtain distribution information and assesses the significance of genes collected in the KEGG category.

### 2.8. Functional Enrichment Analysis of Gene Ontology for Differentially Expressed Genes

Gene Ontology (GO) analysis facilitates the functional annotation of each gene and determines the most significant functions within a specific set of genes through statistical analyses. GO functional enrichment analysis comprises two main steps: initially, GO function annotation utilizes differentially expressed genes to provide annotations for molecular function, biological process, and cellular component, thus providing essential background knowledge for functional classification tags and gene function studies. Subsequently, based on the GO annotations of genes, all genes of the species (chicken) are selected as background genes, and the *p*-value is calculated using the hypergeometric method. A significance threshold of *p* < 0.05 is employed to identify highly frequent annotations with statistical significance relative to the background. This process yields distribution information and significance regarding gene enrichment in the GO category. The selection of enriched GO terms was based on the 10 top terms, ordered by their significance with a descending rank of *p*-values.

### 2.9. Quantitative Real-Time Polymerase Chain Reaction (qRT-PCR)

To validate the accuracy of the RNA-Seq results, we conducted in vitro confirmatory experiments by qRT-PCR. Given the widespread presence of macrophages in the spleen and thymus, we selected the chicken macrophage line (HD11) as the cellular model for validation. HD11 cells were inoculated with Mukteswar and JS/7/05/Ch at 1 MOI for 24 h. Subsequently, total RNA was extracted from the infected cells and synthesized into cDNA. Each sample was then subjected to qRT-PCR to examine the amplification of five randomly selected DEGs: TRAT1, JUP, LPAR4, CYB561A3, and CXCR5. The experimental procedures for qRT-PCR followed published protocols. The primers of qRT-PCR were shown in Table S1.

### 2.10. Statistical Analysis

The GO and KEGG pathway analyses of coexpressed genes were implemented via the R package clusterProfiler (3.14.3) [23]. The *p*-values are calculated using the hypergeometric distribution method (parameter: pvalueCutoff = 0.05, pAdjustMethod = BH, qvalueCutoff = 0.05), and *p* < 0.05 was considered statistically significant for functional enrichment analysis. The statistical significance of TCID_50_, EID_50_, and MDT assays was assessed using Student’s *t* test with GraphPad Prism 7.00 software. In addition, the statistical significance of qPCR was assessed using one- or two-way analysis of variance (ANOVA) with GraphPad Prism 7.00 software. A *p*-value of less than 0.05 was deemed statistically significant.

## 3. Results

### 3.1. Characterization of the Virulence and Pathogenicity of Model Viruses

A pair of model viruses, Mukteswar and JS/7/05/Ch, were diluted and inoculated into CEF cells, SPF chicken embryos, and SPF chickens following the standard procedures for ND, respectively. Genotype III NDV exhibited comparable TCID_50_, EID_50_, and MDT values between the two viruses. The TCID_50_ value for Mukteswar was significantly higher than that of JS/7/05/Ch, likely because JS/7/05/Ch causes more severe cell damage [18], hindering its ability to replicate in cell cultures. The EID_50_ and MDT values for both Mukteswar and JS/7/05/Ch showed comparable results (Figure S1). The ICPI value of JS/7/05/Ch (ICPI: 1.90) was slightly higher than that of Mukteswar (ICPI: 1.58). Notably, the IVPI scores for velogenic NDVs were >3.0, whereas mesogenic strains scored 0.0–0.5, and lentogenic strains scored 0.0 [24,25]. Here, the IVPI value of JS/7/05/Ch was significantly higher than that of Mukteswar following the intravenous inoculation of SPF chickens. Briefly, the IVPI value of JS/7/05/Ch was 1.34, indicating a highly virulent strain, while the IVPI value of Mukteswar was only 0.11, signifying a moderately virulent strain. 

**Figure 2:**
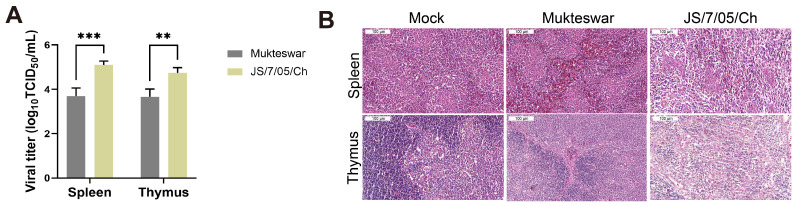
Viral pathogenic characteristics of NDV in vivo. (**A**) Determination of virus load in the spleen and thymus following NDV infection. (**B**) Histopathology of the spleen and thymus following NDV infection. Magnification 200×, scale bar 100 μm. ** *p* < 0.01, *** *p* < 0.001. Bar-plot error bars indicate SDs.

Subsequently, SPF chickens were infected with model viruses at 10^6^ EID_50_/0.1 mL through intravenous injection, and significant differences in pathogenicity appeared 4 days post-infection (dpi). We assessed the viral load in the spleen and thymus of the infected group. TCID_50_ results indicated successful infection by both Mukteswar and JS/7/05/Ch strains in chickens, with JS/7/05/Ch demonstrating notably higher virus replication levels in the spleen and thymus (Figure 2A). The two NDV-infected groups showed histopathological damage compared to the mock group. Specifically, infection with JS/7/05/Ch led to notably severe pathological changes in the spleen and thymus. These changes included extensive inflammatory cell infiltration, areas of necrosis, and the exudation of red blood cells in the spleen. Concurrently, the infected thymus exhibited a significant lymphodepletion and structural damage, indicating a high-intensity impact on the immune system. In contrast, the Mukteswar-infected group displayed only minor histopathological damage. The observed changes were limited to bleeding in both the spleen and thymus (Figure 2B).

### 3.2. Characterization of mRNA Expression Profile Post-NDV Infection in Chickens

**Figure 3:**
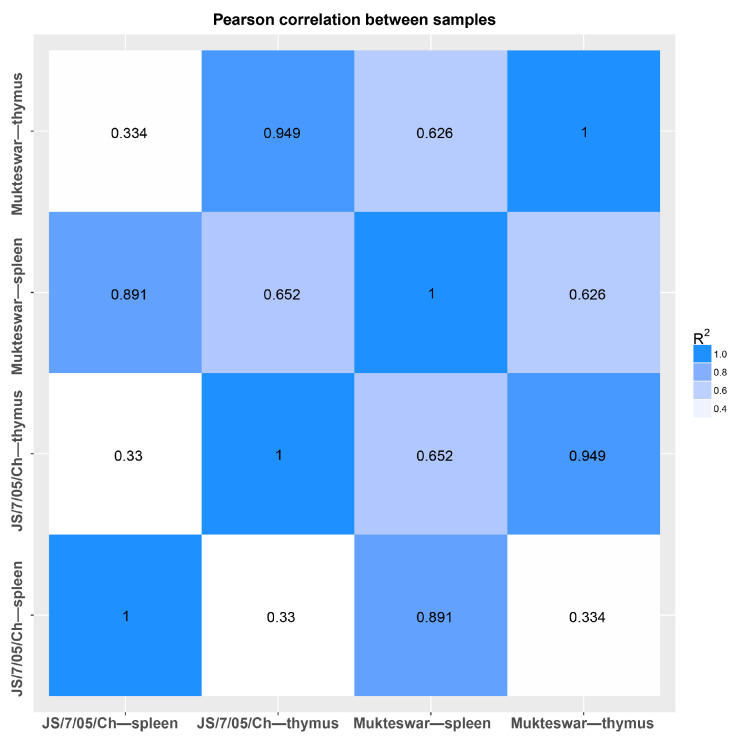
Pearson correlation map between samples.

Mukteswar-inoculated chickens were employed as the control group, while JS/7/05/Ch-inoculated chickens were utilized as the experimental group. For mRNA transcriptome sequencing, the OD260/280 and OD260/230 values for each group met the specified criteria of ≥1.5 and ≥1.0, respectively. The RNA Integrity Number (RIN) exceeded or was equal to 7, satisfying the requirements for animal samples. Both 28S/18S ratios were ≥1, aligning with the integrity criteria for eukaryotic RNA. All test results achieved the highest rating of “A” (Table 1). Furthermore, this study employed the Pearson correlation coefficient to assess the correlation between samples, thereby gauging the reliability of sequencing results. As depicted in Figure 3, the correlation coefficients between the spleen and thymus in each infection group were notably high, reaching 0.891 and 0.949, respectively. Consequently, the samples were deemed qualified and the prerequisites for constructing the second library were fulfilled. 

Following this, the samples underwent processing and sequencing. The quality analysis of the raw data revealed that the total reads for Mukteswar–spleen, Mukteswar–thymus, JS/7/05/Ch–spleen, and JS/7/05/Ch–thymus were 14,775,501, 16,990,362, 15,433,737, and 1,406,476, respectively. Typically, Q20 and Q30 values serve as benchmarks for assessing sequencing quality. The Q20 value signifies that 20% of bases meet or exceed a quality score of 20, corresponding to a maximum error rate of 1%; the Q30 value denotes a stricter standard where at least 30% of bases must score 30 or above, with an error rate of 0.1% or less. These values are critical metrics for evaluating the reliability of sequencing data and the accuracy of subsequent analyses. High Q20 and Q30 values suggest a low error rate in the sequencing data, which is suitable for precise gene expression profiling and variant detection. Generally speaking, the threshold values of Q20 and Q30 exceeding 90% meet the sequencing requirements [26,27]. In this case, the GC content exceeded 46%, with Q20 surpassing 97%, and Q30 exceeding 94%. Subsequent to filtering the raw data, clean data were obtained. Quality statistics for the clean data indicated that the clean reads for Mukteswar-infected spleen/thymus and JS/7/05/Ch-infected spleen/thymus were 14,765,073, 16,979,110, 15,422,587, and 14,056,999, respectively. Q20 was greater than 95%, and Q30 surpassed 99%. These results affirm that the filtered data met the analytical requirements (Table 1). Additionally, due to primer amplification bias, there were significant fluctuations in the 6–10 bases before each read during sequencing. However, the base composition in subsequent cycles tended to stabilize, which is considered a normal phenomenon. Importantly, the base quality for each group consistently exceeded 30 in this study, meeting the analytical requirements (Figures S2 and S3).

**Figure 4:**
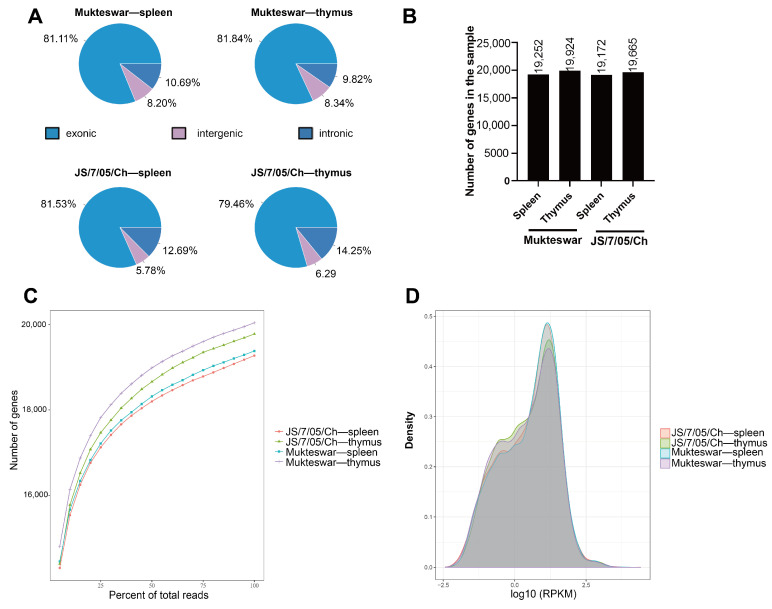
Analysis of read distribution and gene expression. (**A**) Analysis of read distribution. Distribution of reads in gene composition of NDV-infected spleen and thymus. The figures illustrate the proportion of exonic, intronic, and intergenic reads. (**B**) The number of genes in NDV-infected spleen and thymus. (**C**) Evaluation of saturation of sequencing results. The *X*-axis represents the proportion of sequencing reads (expressed as a percentage), while the *Y*-axis indicates the count of identified genes. (**D**) Gene expression density map. Utilizing RPKM density distribution mapping to assess the collective gene expression pattern of the sample, regional statistical analysis of genome-wide gene expression levels was conducted to depict the overall expression profile of the sample.

The chromosomal distribution and gene density exhibited similarities between the Mukteswar- and JS/7/05/Ch-infected groups. Predominantly, the reads were distributed on chromosome 1, followed by chromosomes 2, 3, and 4 (Figure S4). The mapped reads were predominantly located in exons, accounting for approximately 80% (Figure 4A). As the number of reads increased to a specific threshold, the identified genes in each group tended to reach saturation, with the number of genes exceeding 19,000 in each group (Figure 4B). The RPKM method is currently the most widely used approach for estimating gene expression levels, providing an overview of the sample’s overall expression status. In this study, the overall expression density distribution among the groups remained essentially consistent (Figure 4C,D).

### 3.3. Identification of Differentially Expressed Genes

**Figure 5:**
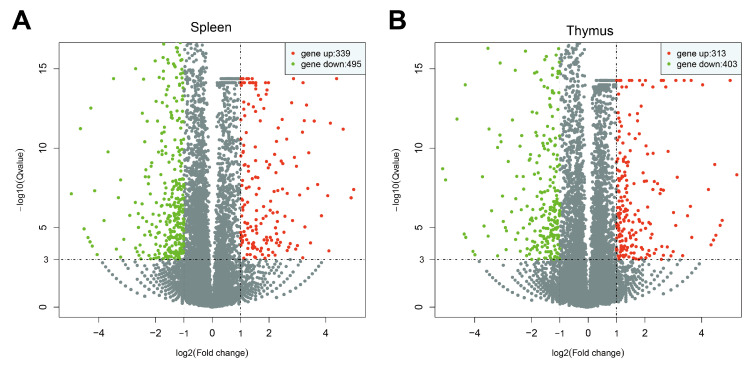
Volcano plot of DEG analysis. (**A**) NDV-infected spleen. (**B**) NDV-infected thymus. The *X*-axis illustrates variations in gene expression multiples across various samples, while the *Y*-axis denotes the statistical significance of disparities in gene expression levels. Red dots indicate genes significantly up-regulated, whereas green dots denote genes significantly down-regulated. Gray dots indicate genes with no significant regulation. The significant differential expression was based on both fold change (|log2FoldChange| > 1) and statistical significance (q-value < 0.001).

We identified genes with significant differential expression based on both fold change (|log2FoldChange| > 1) and statistical significance (q-value < 0.001). A volcano plot was utilized for visual analysis, illustrating the overall distribution of differentially expressed genes. In the spleen, 834 genes exhibited differential expression between the two groups, with 339 up-regulated and 495 down-regulated genes (Figure 5A). In the thymus, 716 genes showed differential expression, including 313 up-regulated and 403 down-regulated genes (Figure 5B). A Venn diagram is a useful method to compare the DEGs across various groups. The Venn results showed that the spleen group exhibited 834 DEGs, while the thymus group showed 716 DEGs, with 190 DEGs common to both (Figure S5).

### 3.4. GO Enrichment Analysis of Differentially Expressed Genes

**Figure 6:**
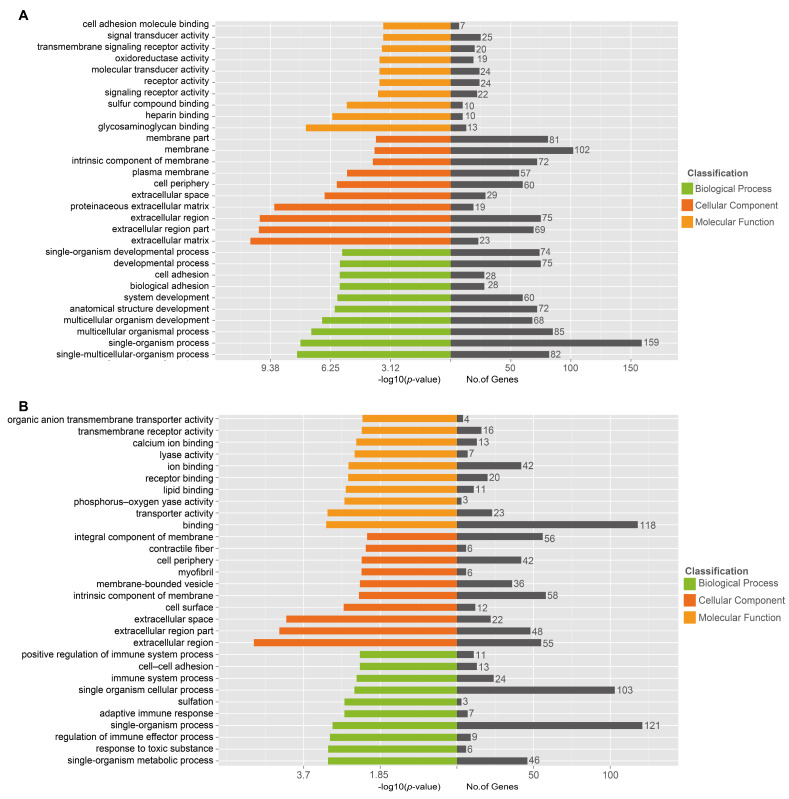
Gene Ontology (GO) functional enrichment of DEGs. The classification map illustrated the GO function enrichment of DEGs in the spleen (**A**) and thymus (**B**) following NDV infection. The *Y*-axis delineates the three fundamental categories of GO (Biological Process, Cellular Component, and Molecular Function) along with their respective specific terms. The *X*-axis indicates the *p*-value associated with each term, alongside the count of genes annotated to that particular term.

To further elucidate the functions of differentially expressed genes during NDV infection in chickens, we conducted Gene Ontology (GO) analysis on host genes exhibiting distinct expression patterns (|log2(FoldChange)| > 1 and q-value < 0.001). In the spleen, the top 10 GO terms showing the highest degree of enrichment in Biological Processes included single-multicellular-organism process, single-organism process, multicellular organismal process, multicellular organism development, anatomical structure development, system development, biological adhesion, cell adhesion, developmental process, and single-organism developmental process. These terms primarily encompassed cell and biological development, as well as the adhesion of cells and organisms. Secondly, the top 10 GO terms exhibiting the highest degree of enrichment in Cellular Component were extracellular matrix, extracellular region part, extracellular region, proteinaceous extracellular matrix, extracellular space, cell periphery, plasma membrane, intrinsic component of membrane, membrane, and membrane part. These terms predominantly included components such as the extracellular part and membrane composition. Thirdly, the top 10 GO terms exhibiting the highest degree of enrichment in Molecular Function included glycosaminoglycan binding, heparin binding, sulfur compound binding, signaling receptor activity, receptor activity, molecular transducer activity, oxidoreductase activity, transmembrane signaling receptor activity, signal transducer activity, and cell adhesion molecule binding. These genes were predominantly expressed in receptor and binding activity (Figure 6A). 

In the thymus, the top 10 GO terms exhibiting the highest degree of enrichment in Biological Process included single-organism metabolic process, response to toxic substance, regulation of immune effector process, single-organism process, adaptive immune response, sulfation, single-organism cellular process, immune system process, cell–cell adhesion, and positive regulation of immune system process. These findings primarily highlight contributions to the development of cellular organisms and immune responses. The top 10 enriched GO terms in Cellular Component included extracellular region, extracellular region part, extracellular space, cell surface, intrinsic component of membrane, membrane-bounded vesicle, myofibril, cell periphery, contractile fiber, and integral component of membrane. This suggests a predominant expression in the extracellular region and membrane composition. Furthermore, the top 10 enriched GO terms in Molecular Function comprised binding, transporter activity, phosphorus–oxygen lyase activity, lipid binding, receptor binding, ion binding, lyase activity, calcium ion binding, transmembrane receptor activity, and organic anion transmembrane transporter activity. This mainly encompasses receptor and binding activity (Figure 6B). When comparing the highly enriched GO terms between the spleen and thymus, it was observed that five GO terms were identical. The spleen and thymus shared commonalities in cell adhesion and development processes, but differed in their specific functional enrichments, with the spleen emphasizing general multicellular development and the thymus highlighting immune response and metabolic processes. Both organs featured extracellular and membrane components, with the spleen’s molecular functions biased towards binding activities, while the thymus showed a unique focus on transporters and transmembrane receptors.

### 3.5. KEGG Enrichment Analysis of Differentially Expressed Genes

**Figure 7:**
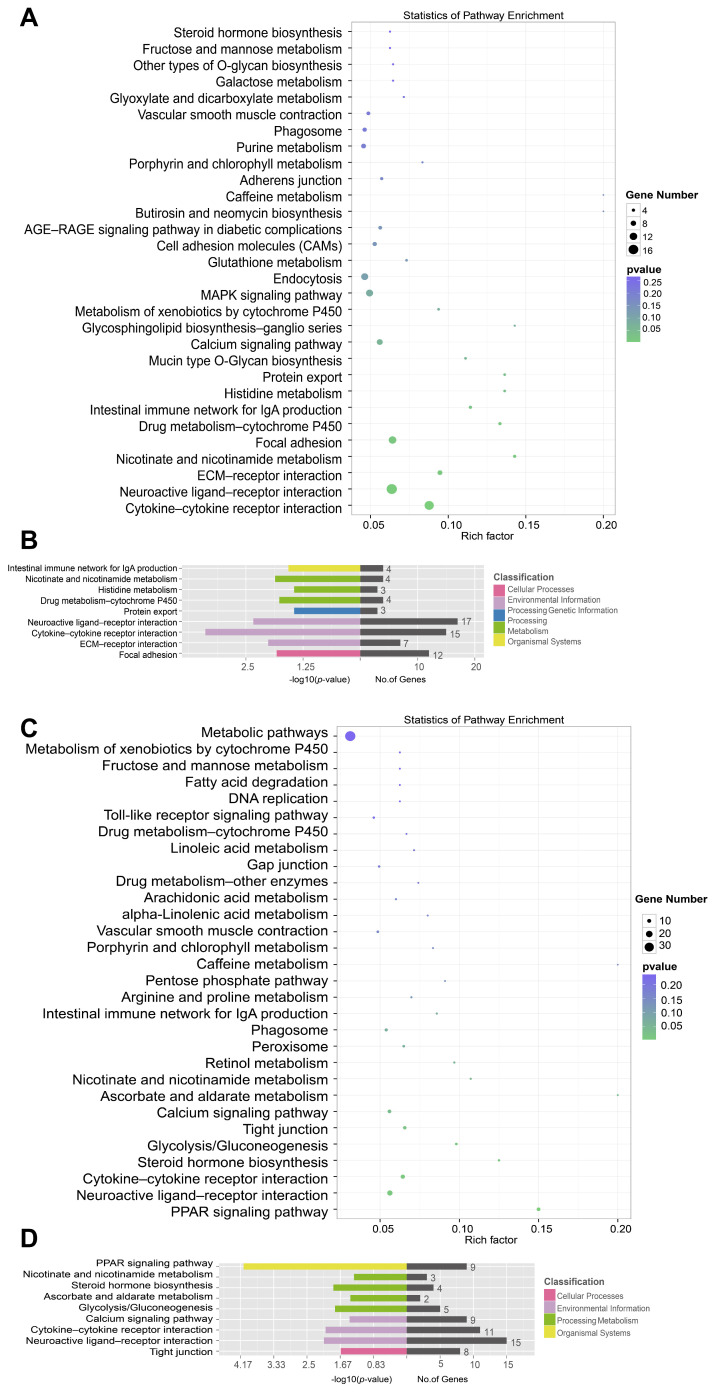
Kyoto Encyclopedia of Genes and Genomes (KEGG) annotation of DEGs. (**A**) KEGG pathway bubble map of DEGs. This visualization presents the statistical enrichment of pathways in NDV-infected spleen. (**B**) KEGG pathway annotation for DEGs in the NDV-infected spleen. (**C**) KEGG pathway bubble map of DEGs. This visualization presents the statistical enrichment of pathways in NDV-infected thymus. (**D**) KEGG pathway annotation for DEGs in the NDV-infected thymus. KEGG pathway bubble map: the *X*-axis denotes the proportion of enriched differential genes within the pathway’s background gene set, while the *Y*-axis lists the pathway names. The size of each bubble represents the number of enriched differential genes, while the color indicates the associated *p*-value. KEGG pathway annotation: the *Y*-axis represents the categories of KEGG pathways, while the *X*-axis indicates the *p*-value of each term along with the number of genes annotated to a particular term.

Initially, KEGG analysis was conducted on genes exhibiting differential expressions (|log2(FoldChange)| > 1 and q-value < 0.001), yielding comprehensive results. In the spleen, a total of 332 differentially expressed genes were identified and enriched in the KEGG database. Among these genes, 134 were up-regulated, associated with 76 pathways, while 198 were down-regulated, associated with 91 pathways. As illustrated in the bubble plot, we identified 30 pathways enriched with differentially expressed genes. These pathways were categorically linked to cellular processes, environmental information processing, genetic information processing, metabolism, and organismal systems. Briefly, the top 10 pathways that exhibited significant enrichment predominantly encompassed cytokine–cytokine receptor interaction, neuroactive ligand–receptor interaction, ECM–receptor interaction, nicotinate and nicotinamide metabolism, focal adhesion, drug metabolism-cytochrome P450, intestinal immune network for IgA production, histidine metabolism, and protein export (Figure 7A,B).

In the thymus, 310 differentially expressed genes were identified and subjected to KEGG analysis. Out of these, 89 genes were up-regulated, contributing to 49 pathways, while 221 genes were down-regulated, contributing to 91 pathways. As shown in the bubble plot, we identified 30 pathways enriched with differentially expressed genes that were associated with cellular processes, environmental information processing, metabolism, and organismal systems. Briefly, the top 10 pathways showing significant enrichment predominantly included PPAR signaling pathway, neuroactive ligand–receptor interaction, cytokine–cytokine receptor interaction, steroid hormone biosynthesis, glycolysis/gluconeogenesis, tight junction, calcium signaling pathway, ascorbate and aldarate metabolism, and nicotinate and nicotinamide metabolism (Figure 7C,D). The spleen and thymus exhibited both shared and distinct KEGG pathway enrichments among differentially expressed genes. Commonalities included pathways related to cytokine–cytokine receptor interaction, neuroactive ligand–receptor interaction, and metabolism, such as nicotinate and nicotinamide metabolism. However, the spleen specifically highlighted pathways like intestinal immune network for IgA production, protein export, ECM–receptor interaction, focal adhesion, histidine metabolism, and drug metabolism-cytochrome P450, while the thymus was unique in its enrichment of PPAR signaling pathway, steroid hormone biosynthesis, ascorbate and aldarate metabolism, glycolysis/gluconeogenesis, tight junction, and calcium signaling pathway. Both organs also showed enrichment in pathways associated with cellular processes and environmental information processing, but with different specific pathways within these categories.

### 3.6. Validation of RNA-Seq Data by Quantitative Real-Time PCR (qRT-PCR)

**Figure 8:**
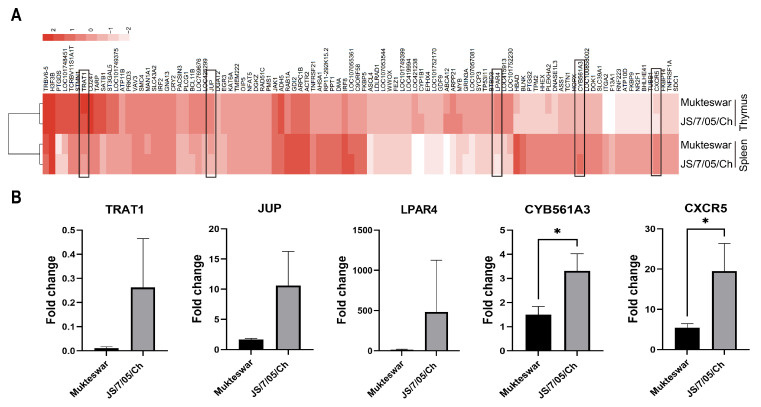
Screening and identification of DEGs. (**A**) A heatmap of the top 100 DEGs was generated in the R package heatmap. (**B**) Expression levels of five candidate genes were assessed. The relative expression of genes randomly selected from the list of candidates was detected using RT-qPCR. * *p* < 0.05. Bar-plot error bars indicate SDs.

We employed the R package heatmap to generate a heatmap of the top DEGs. Several DEGs were identified between the JS/7/05/Ch and Mukteswar groups. In essence, the significant DEGs in spleens infected with JS/7/05/Ch versus Mukteswar encompassed TRBV6-5, PTGDS, TRAT1, MAN1A1, BCL11B, JUP, EGR3, GRIN3A, LPAR4, CYB561A3, and two uncharacterized genes, LOC101748451 and LOC107055361. For thymus infections, the DEGs included TRAT1, CYP1B1, ABCA12, MYB, HBAD, PLEKHA2, and CXCR5. The TRAT1 gene was identified as a common DGE in both the spleen and thymus (Figure 8A). To further verify the RNA-seq data, we chose a subset of five DGEs from RNA-seq statistical analysis for qRT-PCR analysis. The qRT-PCR revealed noticeable differences in the expression levels of TRAT1, JUP, LPAR4, CYB561A3, and CXCR5 between the Mukteswar- and JS/7/05/Ch-infected groups. Particularly, JS/7/05/Ch elicited higher expression levels of these DGEs compared to Mukteswar, with significant differences observed for CYB561A3 and CXCR5 (Figure 8B). These results bolster the reliability of the identified DGEs through RNA-Seq.

## 4. Discussion

The control of ND involves the strict implementation of biosecurity measures to prevent the introduction of virulent NDV onto poultry farms, as well as proper administration of vaccines [28]. The ND vaccine is widely utilized in poultry farming across several countries, including China. Mukteswar, a classical I ND vaccine strain, is commonly employed to boost immunity in poultry aged over two months. However, RNA viruses are susceptible to mutation, leading to changes in viral virulence due to their lack of proofreading activity and the high immune pressure they face [17]. Previous research has indicated that Mukteswar can evolve into a virulent NDV strain known as JS/7/05/Ch during poultry vaccination. This vaccine variant, JS/7/05/Ch, shares high genomic similarity with Mukteswar, with major amino acid mutations primarily concentrated in the HN protein [16]. The HN protein is recognized for its multifunctional role in various biological activities. Our prior study has demonstrated that the mutant HN protein of JS/7/05/Ch significantly impacts viral virulence and biological activities both in vitro and in vivo [18]. However, the mechanisms underlying the regulation of host intrinsic responses by viral infection remain unclear.

Currently, high-throughput sequencing technology has emerged as a potent method for detecting pathogens [29]. High-throughput sequencing has gradually unveiled the pathogenic mechanism of NDV. For instance, Zhang et al. [30] employed transcriptome analysis to identify DEGs between two inbred chicken lines. These DEGs and their associated pathways were predominantly linked to immune response, EIF-signaling, actin cytoskeleton organization nitric oxide production, and coagulation system. The findings offer insights into the distinct responses to NDV observed between the two chicken lines. Virulent NDV infection can cause severe tissue damage in immune organs. Several researchers have explored the regulation of NDV infection within immune organs using high-throughput sequencing technology. For example, Hu et al. [31] successfully illustrated that genotype VII NDV strains induced a robust cytokine response in immune organs through cytokine gene expression profiling. This heightened response may be correlated with elevated levels of virus replication and an intense inflammatory response. Guo et al. [32] investigated the altered expression of immune-related genes in chicken thymus following NDV infection using RNA-seq. Their results revealed significant up-regulation of AvBD5, IL16, IL22, and IL18R1, which play pivotal roles in defense against NDV. Additionally, Il-18 expression exhibited changes, albeit not statistically significant. These findings collectively highlight the importance of RNA-seq studies in elucidating the underlying mechanisms driving the enhancement of NDV virulence.

In this study, RNA-seq analyses were conducted on chicken spleen and thymus following NDV infection. The results revealed a significant number of DEGs between two NDV-infected groups, including 834 DEGs in spleen and 716 DEGs in thymus. To comprehensively elucidate the biological functions of these DEGs, we initially performed GO enrichment analysis on these genes. The results of the enrichment analysis in Biological Processes highlighted a predominant involvement of binding and receptor activities in both the spleen and thymus. This finding aligns with our previously reported observation that these two NDV strains elicited diverse receptor binding activities attributable to the mutant HN protein [18]. The enrichment analysis of Cellular Component revealed a predominant focus on extracellular and membrane components in both the spleen and thymus. This suggests that Mukteswar and JS/7/05/Ch could exert notable distinct regulatory effects on cellular membranes and peripheries. Meanwhile, there may exist differences in viral function at the cell surface. Intriguingly, our prior study has documented that JS/7/05/Ch demonstrates heightened activities in adsorption, internalization, and release compared to Mukteswar, all of which primarily manifest around the cell surface [20]. The enrichment results in Molecular Function indicated a predominant focus on organismal process/development, immune process, and adhesion in the spleen and thymus. These findings indicate a substantial discrepancy in the immune response between JS/7/05/Ch- and Mukteswar-infected chickens, potentially accounting for the divergent pathogenicity of the viruses. Subsequently, we conducted KEGG enrichment analysis to assess these DEGs. The significantly enriched pathways in the spleen and thymus predominantly involved molecule–receptor interaction and metabolism. This observation implied that Mukteswar and JS/7/05/Ch may manifest distinct virulence by modulating host molecule–receptor interactions and metabolic processes differentially. Particularly noteworthy is the enrichment of DEGs in cytokine-related pathways, aligning with our prior observations that JS/7/05/Ch elicited a more pronounced inflammatory response compared to Mukteswar [16]. Generally, increased levels of inflammatory cytokines contribute to the acute cytokine storm syndrome, underpinning the high pathogenicity of virulent NDV [33]. Moreover, the enriched pathways encompassed immune- and junction-related pathways. Immunity is the main host defense against NDV infection [34]; meanwhile, cellular junctions can affect viral infection, such as tight junction and adhesive junction [35,36]. These findings suggest that the DEGs with immune and junction functions may play significant roles in diverse pathogenicity between JS/7/05/Ch and Mukteswar. 

We employed the R package heatmap to generate a heatmap of the top DEGs, and screened several DEGs including TRBV6-5, PTGDS, TRAT1, MAN1A1, BCL11B, JUP, EGR3, GRIN3A, LPAR4, and CYB561A3 in spleen and TRAT1, CYP1B1, ABCA12, MYB, HBAD, PLEKHA2, and CXCR5 in thymus. Based on the published functions of these genes, we have compiled a summary. TRBV6-5 and BCL11B are primarily implicated in the immune response post-infection [37,38]; PTGDS, EGR3, and CXCR5 modulate the host’s inflammatory response [39,40,41]. Here, JS/7/05/Ch and Mukteswar have been demonstrated to induce differential immune levels and inflammatory responses [16], and it is worth exploring the roles played by these genes in this effect. CYB561A3, LPAR4, CYP1B1, and HBAD contribute to cellular metabolism, affecting cell development and proliferation [42,43,44,45]. JS/7/05/Ch has been demonstrated to cause severe cell death compared to Mukteswar [19]; these genes are suspected to participate in the NDV-mediated differential cell death pathway. TRAT1 and MAN1A1 are key factors in protein synthesis and modification, vital for protein function [46,47]. Differentiation between JS/7/05/Ch and Mukteswar primarily lies in the HN protein, with previously reported disparities in their HN protein functions [18], suggesting that these DEGs may influence the biological function of the HN protein. JUP and PLEKHA2, associated with cellular cytoskeleton structure [48,49], align with our prior findings on cytoskeletal protein vimentin’s role in NDV infection [20]. This finding suggests these structural proteins as promising research targets; the potential interaction among these cellular structural proteins will be a subject of interest. MYB is a NF-kB direct target gene, encoding a transcriptional activator whose loss leads to apoptosis [50,51]. Our previous study demonstrates that JS/7/05/Ch and Mukteswar differentially activate NF-kB, leading to distinct cell apoptosis and virus replication [19]. The role of MYB in this mechanism will be a subject for future investigation. GRIN3A is considered as a neuroregulatory gene [52]. The highly virulent NDV JS/7/05/Ch induces neurological symptoms [18], warranting exploration of its virulence correlation with GRIN3A. ABCA12 has been shown to be a lipid transporter [53], and viruses require host lipids for their transport through the membranes [54]. Therefore, the role of ABCA12 in modulating NDV infection and virulence through the regulation of lipid remains to be elucidated. Overall, the RNA-seq-identified DEGs, known for their roles in viral processes, warrant systematic study to determine their functional impact on NDV infection and virulence. In addition, the random selective qPCR validation of these DEGs revealed differences between the Mukteswar- and JS/7/05/Ch-infected groups, further confirming the accuracy of the sequencing results. Significantly, CYB561A3 and CXCR5 exhibit pronounced differential expression. CYB561A3 is a crucial gene involved in endosomal and lysosomal cellular iron homeostasis [42]. Here, JS/7/05/Ch significantly up-regulated mRNA levels of CYB561A3 compared to Mukteswar, indicating their distinct impacts on CYB561A3 function. Considering its important role in cellular iron homeostasis, we will investigate the interaction between CYB561A3 and ferroptosis during NDV infection in the future. Furthermore, JS/7/05/Ch also induced higher expression levels of CXCR5, a cytokine receptor [55], compared to Mukteswar. Our results indicate that JS/7/05/Ch may enhance inflammatory response by targeting the CXCR5 gene; excessive inflammatory response may result in higher virulence and pathogenicity. Therefore, transcriptome sequencing yields insights for future research, which are speculative and await experimental validation.

## 5. Conclusions

This study offers a comprehensive insight into the differential changes in transcription levels in chicken spleen and thymus following infection with Mukteswar and its variant virulent NDV strain. The results advance our comprehension of the mechanisms underlying virulence enhancement and offer novel targets for antiviral treatment strategies against NDV.

## Figures and Tables

**Table 1:** The quality information of sequencing sample, raw data, and clean data.

Sample Name	K5500		Agilent 2200		Test Summary	Total Reads	Clean Reads	GC(%)	Raw		Clean	
	OD260/280	OD260/230	RIN	28S/18S					Q20(%)	Q30(%)	Q20(%)	Q30(%)
Mukteswar–spleen	2.03	2.27	7.9	2.1	A	14,775,501	14,765,073	48.55	97.35	95.03	95.10	99.79
Mukteswar–thymus	2.01	1.82	9.9	1.6	A	16,990,362	16,979,110	47.69	97.31	94.95	95.10	99.83
JS/7/05/Ch–spleen	2.01	2.08	9.8	2.2	A	15,433,737	15,422,587	47.61	97.34	95.02	95.11	99.80
JS/7/05/Ch–thymus	2.02	2.23	10.0	2.5	A	14,067,476	14,056,999	46.90	97.35	95.03	95.04	99.81

## Data Availability

Data associated with this article are included in the manuscript and Supplemental Materials.

## Supplementary Materials

The following supporting information can be downloaded at: https://www.mdpi.com/article/10.3390/vetsci11110569/s1, Figure S1. Characterization of TCID_50_, EID_50_, MDT values of NDV. * *p* < 0.05; ns, no significance. Bar-plot error bars indicate SDs. Figure S2. Base composition distribution map of clean reads. (A) Mukteswar-infected spleen. (B) Mukteswar-infected thymus. (C) JS/7/05/Ch-infected spleen. (D) JS/7/05/Ch-infected thymus. Figure S3. Base mass distribution map of raw reads. (A) Mukteswar-infected spleen. (B) Mukteswar-infected thymus. (C) JS/7/05/Ch-infected spleen. (D) JS/7/05/Ch-infected thymus. Figure S4. Distribution of reads on chromosomes. (A) Mukteswar-infected spleen. (B) Mukteswar-infected thymus. (C) JS/7/05/Ch-infected spleen. (D) JS/7/05/Ch-infected thymus. The *X*-axis depicts chromosome positions, while the *Y*-axis represents read abundance. Green indicates the positive strand, while yellow indicates the negative strand. Figure S5. Venn diagram of DEGs between infected spleens and thymuses. Table S1. Primer sequences used in this study. Table S2. Raw gene data of spleen. Table S3. Raw gene data of thymus.
