# Supplementary material for: RNA-Seq Profiling in Chicken Spleen and Thymus Infected with Newcastle Disease Virus of Varying Virulence
Source: Vet Sci. 2024 Nov 15;11(11):569. doi: 10.3390/vetsci11110569 (PMC11599091; doi:10.3390/vetsci11110569)
Supplement: Supplementary file 1 [file vetsci-11-00569-s001.zip › Supplementary materials/Table S1.docx]

**Table S1 Primer sequences used in this study**

| **Gene name** | **Primer sequence (5’-3’)** |
| --- | --- |
| TRAT1-F | CGGCAACTCCGGAGATAGATT |
| TRAT1-R | CTTCCAGTGGCCATCCTAGT |
| JUP-F | GACCTCATGAACCGCATGGA |
| JUP-R | GTGAGTAGAGGAGCTGCACG |
| LPAR4-F | GAGGGCTGATACTGAAGGATGAA |
| LPAR4-R | CAGGAAGGTCGCTTGAACCC |
| CYB561A3-F | AGGAGGGGTTGAGGTCCC |
| CYB561A3-R | AAGGTGCAGAAAGGCAGGAA |
| CXCR5-F | GACCTATGACTTGAGCCTGGT |
| CXCR5-R | GAAGGGGTCTCTCTGGTTGC |
| β-actin-F | ATTGTCCACCGCAAATGCTTC |
| β-actin-R | AAATAAAGCCATGCCAATCTCGTC |
