# Supplementary figures and images for: RNA-Seq Profiling in Chicken Spleen and Thymus Infected with Newcastle Disease Virus of Varying Virulence
Source: Vet Sci. 2024 Nov 15;11(11):569. doi: 10.3390/vetsci11110569 (PMC11599091; doi:10.3390/vetsci11110569)

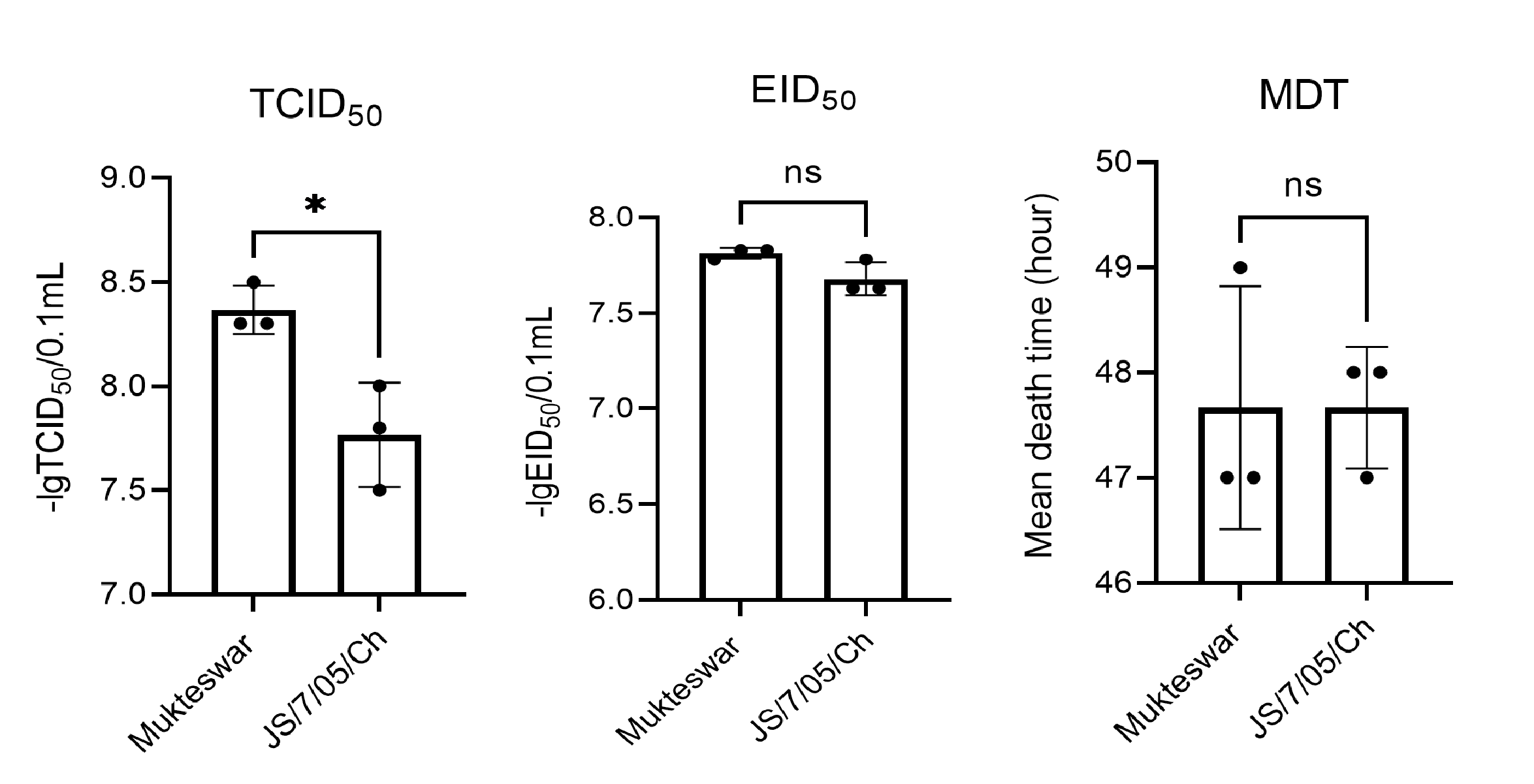

Supplement: Supplementary file 1 [file vetsci-11-00569-s001.zip › Supplementary materials/S1 Fig.tif]

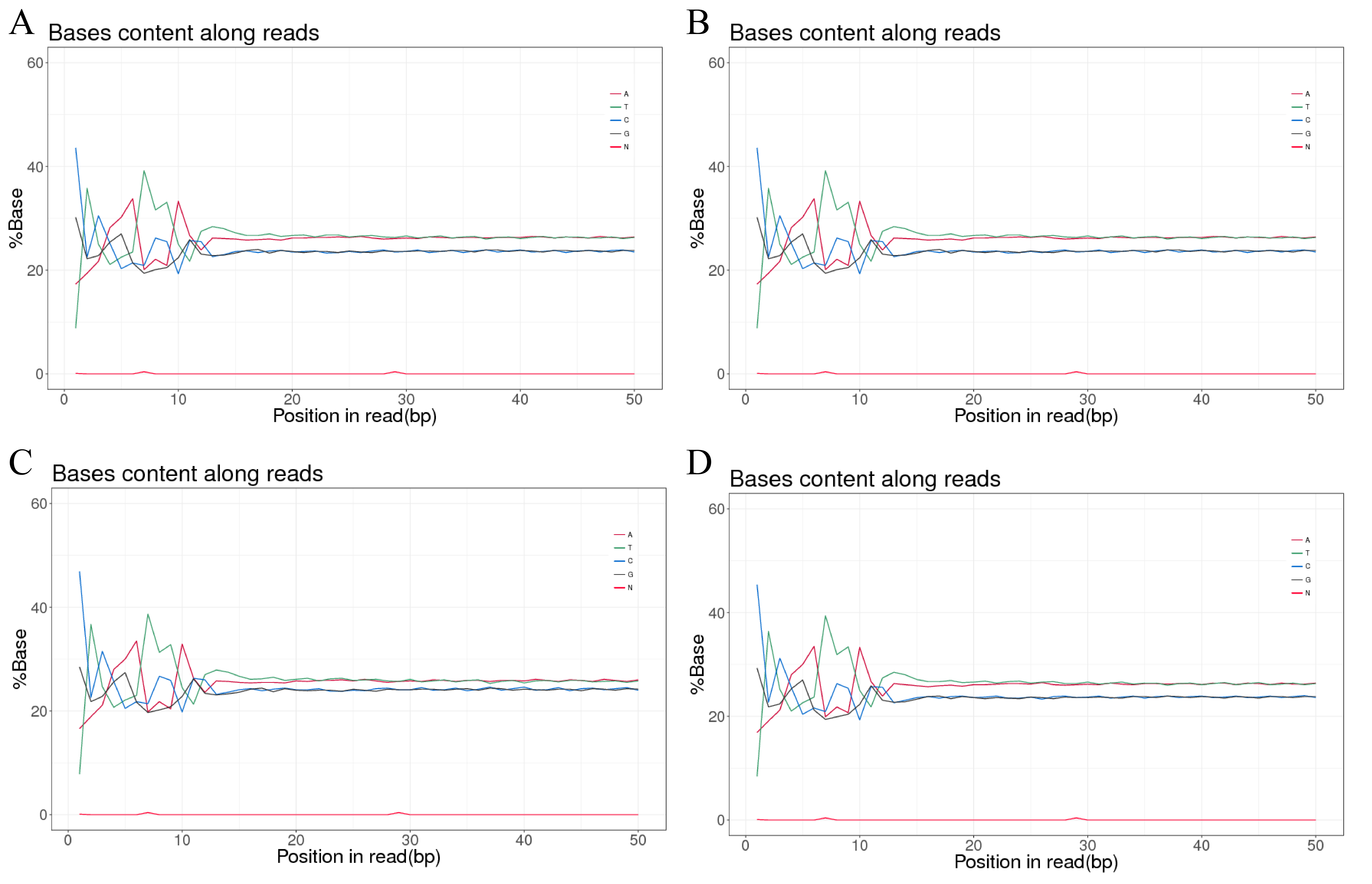

Supplement: Supplementary file 1 [file vetsci-11-00569-s001.zip › Supplementary materials/S2 Fig.tif]

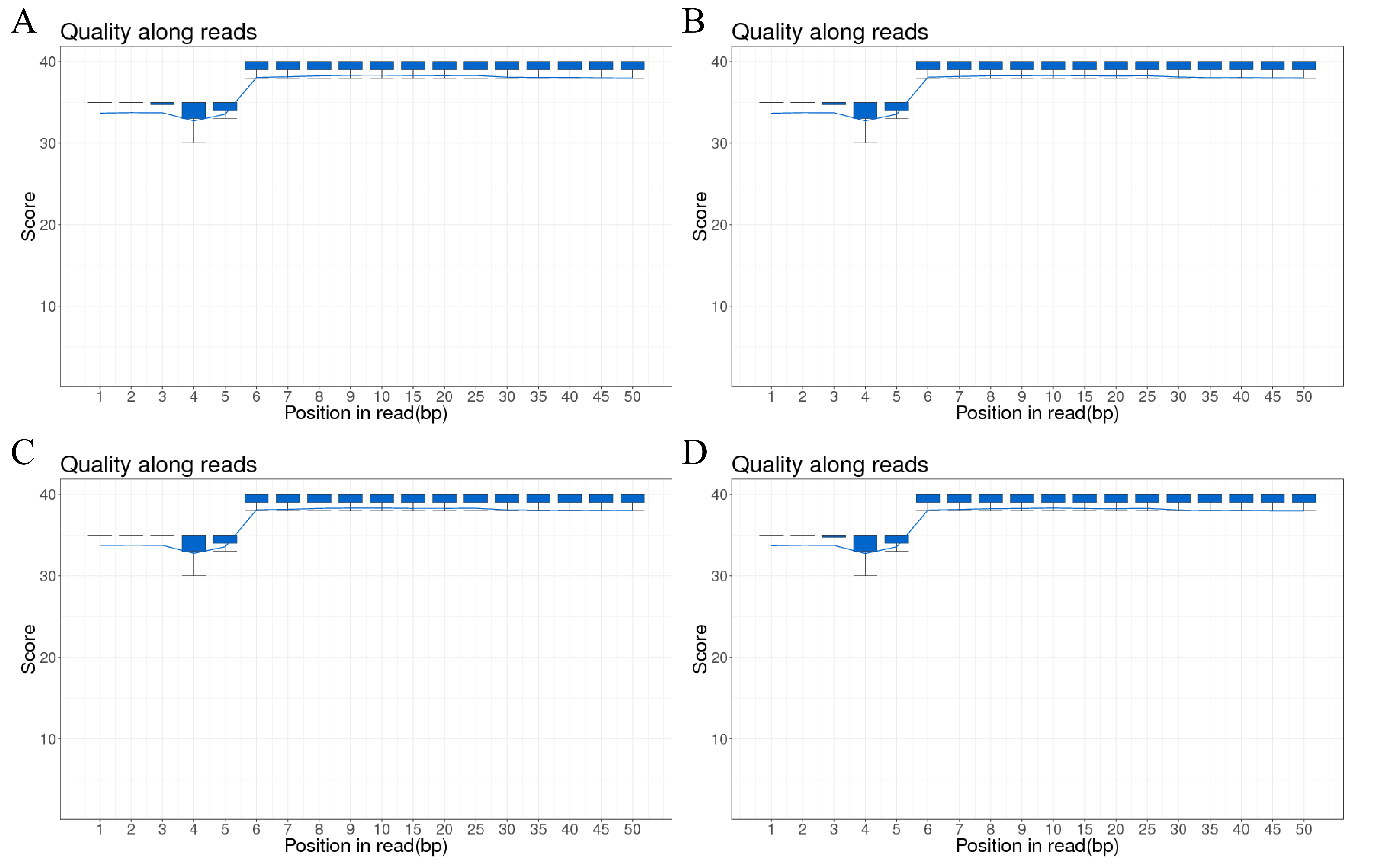

Supplement: Supplementary file 1 [file vetsci-11-00569-s001.zip › Supplementary materials/S3 Fig.tif]

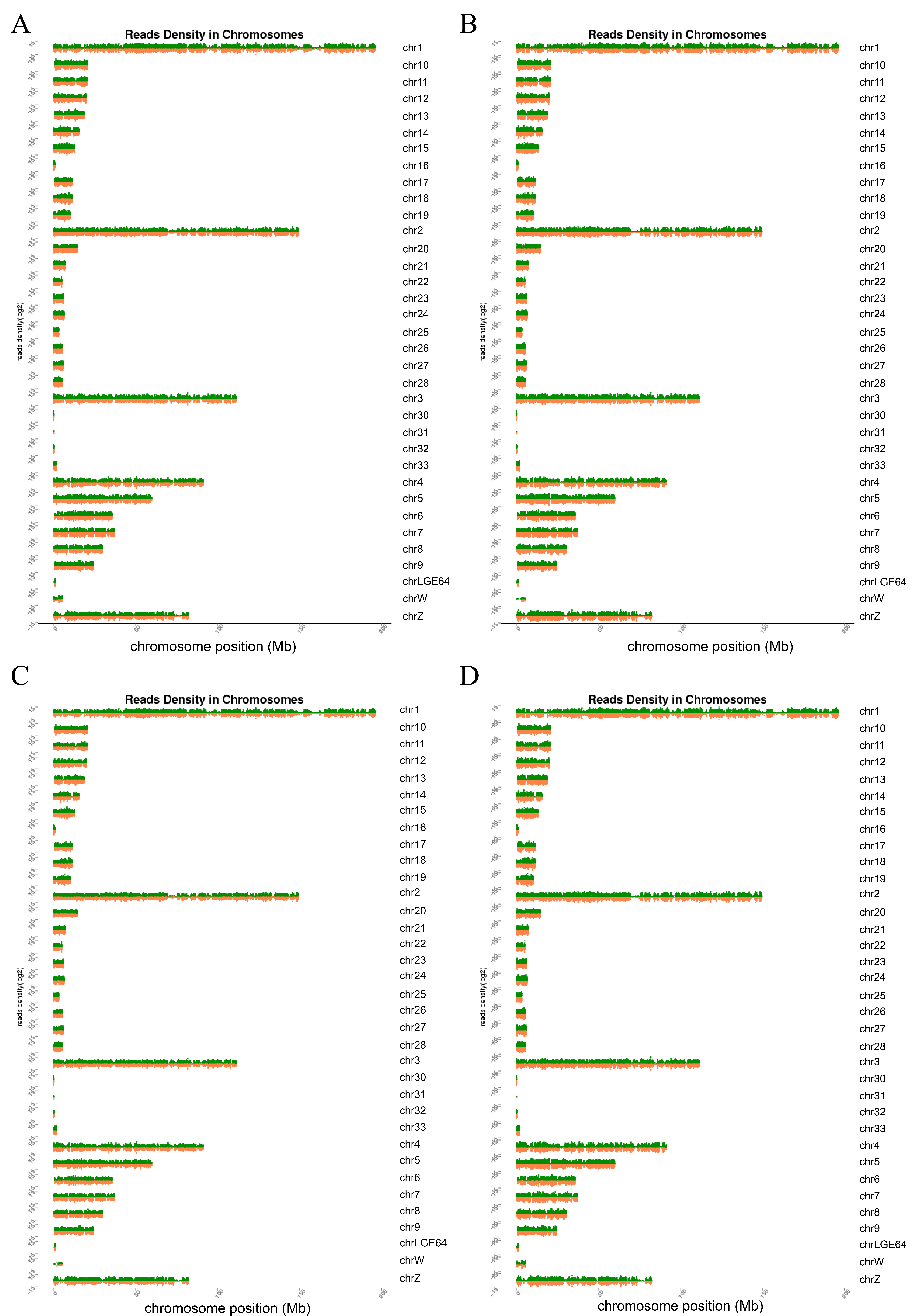

Supplement: Supplementary file 1 [file vetsci-11-00569-s001.zip › Supplementary materials/S4 Fig.tif]

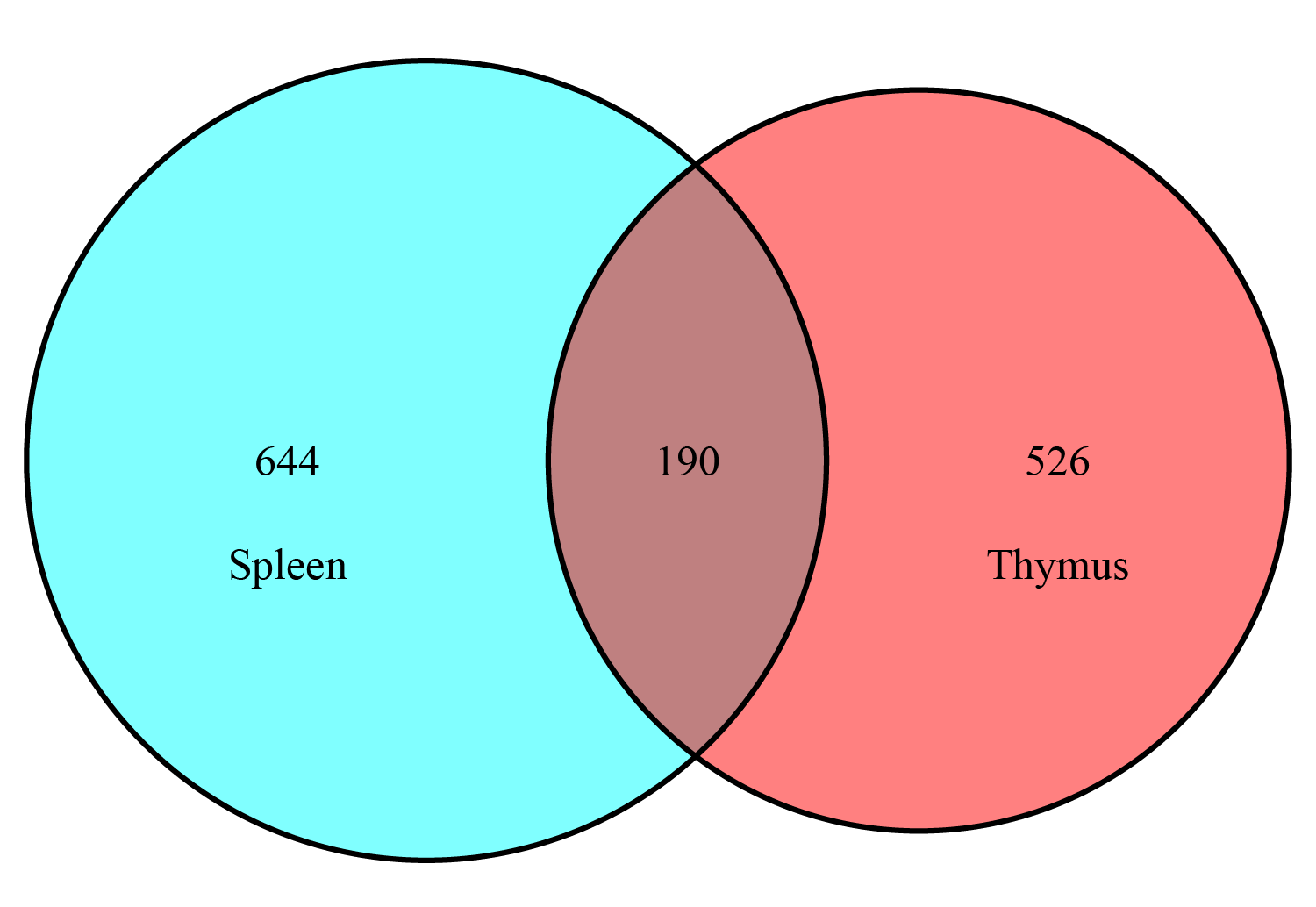

Supplement: Supplementary file 1 [file vetsci-11-00569-s001.zip › Supplementary materials/S5 Fig.tif]
